# Investigation of G72 (DAOA) expression in the human brain

**DOI:** 10.1186/1471-244X-8-94

**Published:** 2008-12-11

**Authors:** Isabel Benzel, James NC Kew, Ramya Viknaraja, Fiona Kelly, Jacqueline de Belleroche, Steven Hirsch, Thirza H Sanderson, Peter R Maycox

**Affiliations:** 1 Psychiatry, GlaxoSmithKline, New Frontiers Science Park, Harlow, Essex, CM19 5AW, UK; 2 Biopharm Discovery Technology Group, GlaxoSmithKline, Stevenage, Hertfordshire, UK; 3 Bioinformatics, GlaxoSmithKline, Harlow, Essex, UK; 4 Core Discovery Technology Group, GlaxoSmithKline, New Frontiers Science Park, Harlow, Essex, CM19 5AW, UK; 5 Division of Neuroscience and Mental Health, Faculty of Medicine, Imperial College London, Hammersmith Hospital, London, UK

## Abstract

**Background:**

Polymorphisms at the G72/G30 locus on chromosome 13q have been associated with schizophrenia or bipolar disorder in more than ten independent studies. Even though the genetic findings are very robust, the physiological role of the predicted G72 protein has thus far not been resolved. Initial reports suggested G72 as an activator of D-amino acid oxidase (DAO), supporting the glutamate dysfunction hypothesis of schizophrenia. However, these findings have subsequently not been reproduced and reports of endogenous human G72 mRNA and protein expression are extremely limited. In order to better understand the function of this putative schizophrenia susceptibility gene, we attempted to demonstrate G72 mRNA and protein expression in relevant human brain regions.

**Methods:**

The expression of G72 mRNA was studied by northern blotting and semi-quantitative SYBR-Green and Taqman RT-PCR. Protein expression in human tissue lysates was investigated by western blotting using two custom-made specific anti-G72 peptide antibodies. An in-depth *in silico *analysis of the G72/G30 locus was performed in order to try and identify motifs or regulatory elements that provide insight to G72 mRNA expression and transcript stability.

**Results:**

Despite using highly sensitive techniques, we failed to identify significant levels of G72 mRNA in a variety of human tissues (e.g. adult brain, amygdala, caudate nucleus, fetal brain, spinal cord and testis) human cell lines or schizophrenia/control post mortem BA10 samples. Furthermore, using western blotting in combination with sensitive detection methods, we were also unable to detect G72 protein in a number of human brain regions (including cerebellum and amygdala), spinal cord or testis. A detailed *in silico *analysis provides several lines of evidence that support the apparent low or absent expression of G72.

**Conclusion:**

Our results suggest that native G72 protein is not normally present in the tissues that we analysed in this study. We also conclude that the lack of demonstrable G72 expression in relevant brain regions does not support a role for G72 in modulation of DAO activity and the pathology of schizophrenia via a DAO-mediated mechanism. *In silico *analysis suggests that G72 is not robustly expressed and that the transcript is potentially labile. Further studies are required to understand the significance of the G72/30 locus to schizophrenia.

## Background

G72 and G30 are overlapping genes transcribed from opposite strands on chromosome 13q33. They were initially identified within a 65 kb region containing markers associated with schizophrenia in two independent disease cohorts [1]. Genetic association between the G72/G30 locus and both schizophrenia and bipolar disorder has subsequently been reported in several studies and is supported by a recent meta-analysis [2]. Thus, the G72/G30 locus may represent a common susceptibility region for both disorders. Whilst the combined evidence for association with both disorders can be considered robust, associated alleles are not consistent across studies and there may not be a distinct haplotype correlated with disease susceptibility [2].

Multiple transcripts for each gene have been cloned by reverse transcription-PCR from human brain, spinal cord and testis cDNA libraries. The longest G72 open reading frame is predicted to encode a putative 153 amino acid protein, isolated from amygdala, caudate nucleus, spinal cord and testis libraries [1]. No homology was found between any of the putative protein products from the transcripts of either gene and any known protein. Putative LG72 orthologues were identified *in silico *in rhesus monkey and ape genomes leading to the suggestion that it might represent a rapidly evolving primate-specific gene. *In vitro *transcription/translation assays with the cloned G72 and G30 candidate transcripts yielded a protein product for LG72 only, which generated a 24-kDa translation product (pLG72) that was reported to localise at the Golgi apparatus in transiently transfected cells [1]. A more recent study, however, demonstrated mitochondrial localisation of overexpressed G72 [3]. Yeast two-hybrid screening identified D-amino acid oxidase (DAO) as a putative protein interactor of pLG72 and recombinant pLG72 was demonstrated to behave as an activator of DAO *in vitro *[1]. On the basis of this observation, which has subsequently not been reproduced [3], G72 has been renamed D-amino acid oxidase activator (DAOA). DAO catabolises D-amino acids including D-serine which is a co-agonist at the N-methyl-D-aspartate (NMDA) receptor [4]. As NMDA receptor hypofunction has been implicated in the pathophysiology of schizophrenia, the report of a functional interaction between G72 and DAO suggests a pathway whereby G72 could modulate DAO activity, D-serine levels and NMDA receptor activity and, thus, contribute to the disease pathology.

To date, however, there have been no convincing reports of expression of native G72 (or G30) protein and accordingly no confirmation of the G72-DAO interaction *in situ*. Moreover, reports of native G72/G30 mRNA expression are limited. In addition to the original study by Chumakov *et al*. [1], cDNAs have also been amplified from human and testis libraries by Hattori *et al*. [5] although only after ≥ 40 cycles of PCR. Korostishevsky *et al*. [6] have subsequently utilised real-time PCR to detect and quantify G72 and G30 mRNA in human post-mortem dorsolateral prefrontal cortex, reporting a tendency towards overexpression of G72 but not G30 transcripts in schizophrenic versus control brain. In order to learn more about the potential role of this schizophrenia susceptibility gene, we have analysed the gene structure and genomic context of G72/G30 and investigated expression of G72 in human tissues at the mRNA and protein level.

## Methods

### Cloning of human G72

Full-length human G72 cDNA (identical to published GenBank sequence AY138546, which results in the longest predicted protein) was amplified from human testis Marathon cDNA (Clontech) by nested PCR with forward primer 5'-GACCCAAAATGCTGGAAAAGCTGAT-3' and reverse primer 5'-CATCAGAAGGATTGGCTGGGAAGAAT-3' and cloned into pCDNA3.2/GW/D-TOPO. In order to obtain N-terminally epitope-tagged G72 in mammalian expression vectors, G72 cDNA was PCR-amplified from above template vector with forward primer 5'-CACCATGCTGGAAAAGCTGATGGGTGC-3' and reverse primer 5'-TCATTCAGCTTTGGTAGAAGTTATTTCCTTGTGG-3'. The PCR product was cloned into pENTR/D-TOPO entry vector using pENTR Directional TOPO Cloning Kit (Invitrogen), following manufacturer's instructions. Resulting entry clones were used in LR recombination reactions (Invitrogen) with pDEST26 (Invitrogen) and pDEST12.2-FLAG (kind gift from Jan Kopf) Gateway destination vector, to obtain G72 cDNA in frame with the respective N-terminal epitope tag (6 × His or FLAG-tag).

### Cell culture and transient transfection

HEK-293 cells were cultured in Minimum Essential Medium (MEM, Gibco) supplemented with 10% fetal calf serum and non-essential amino acids (Gibco) at 37°C and 10% CO_2_. Cells were transiently transfected using Fugene-6 (Roche) according to the manufacturer's instructions. Cells were plated one day before transfection at 4 × 10^6 ^cells per 10 cm-plate, transfected with 5–7 μg plasmid DNA and incubated with transfection mix (complex of plasmid-DNA and Fugene 6 at a ratio of 1:3) for ~40 h prior to preparation of protein lysates.

### RNA isolation and Northern Analysis of G72 mRNA

Total RNA was isolated from HEK-293 cells (mock-transfected or overexpressing His-G72) using the Qiagen RNeasy protocol, according to manufacturer's instructions. The optional on-column DNase digestion step was performed using the Qiagen RNase-free DNase set. RNA was eluted in 40 μl of RNAse-free water and quantified using a spectrophotometer.

For northern gels, 10 μg total RNA in NorthernMax Formaldehyde sample buffer (Ambion) were loaded per lane. Northern blotting was performed as described in "Promega protocols and applications guide", third edition (1996). Briefly, a formaldehyde-containing 1.2% agarose gel was prepared in 1× MOPS buffer. The gel was run in 1× MOPS buffer at 5 V/cm with regular buffer mixing. After completion, the gel was washed three times for 10 min in RNAse-free dH_2_O, soaked 15 min in 0.05 M NaOH, neutralised 15 min in 100 mM Tris-HCl pH 7.5 and soaked for 30 min in 20× SSC before capillary transfer (over-night) onto Hybond-N+ nylon membrane (Amersham). RNA was UV-crosslinked to the membrane (UV Stratalinker) and dried for at least 2 h prior to further use.

Northern blots were probed with a 390 bp NotI-XbaI fragment of G72 cDNA (corresponding to G72 clone AY138546 cDNA without the last 3'-terminal 85 nucleotides) or with beta-actin control probe (BD Biosciences). Probes were ^32^P-dCTP-labelled using Rediprime II Random Prime Labelling System (Amersham) according to manufacturer's instructions and purified with spin columns (Amersham). Prehybridisation and probe hybridisation were performed with Rapid-hyb buffer (Amersham) at 70°C, according to manufacturer's instructions. Stringency washes were performed according to the Rapid-hyb protocol (Amersham). Briefly, membranes were washed for 20 min at room temperature in washing buffer 1 (2× SSC, 0.1% (w/v) SDS) and twice for 15 min at 65°C in washing buffer 2 (0.1× SSC, 0.1% (w/v) SDS). Signals were detected using a sensitive phosphorimager (Typhoon Trio, Amersham), with exposure times between 2 h and over-night.

Alternatively, commercial human brain multiple tissue Northern (MTN) blots (Clontech/BD Biosciences MTN blot II and blot V) were used for analysis of G72 expression in human brain. Blots (nylon membrane) contained approximately 2 μg of poly A+ RNA per lane from eight (MTN blot II) or six (MTN blot V) different brain regions. Hybridisation conditions were essentially as described above, but less stringent conditions (washing buffer 2 at 55°C instead of 65°C) and longer exposure times of up to three days (phosphorimager, Typhoon Trio, Amersham) were used in order to detect even weak signals. Following G72 probing, membranes were stripped with boiling 0.5% SDS and reprobed with a beta-actin-probe to test for equal loading and good RNA quality.

Alternatively to radioactive labelling, northern blots were probed with biotinylated dCTP, using the "SpotLight Random Primer Labeling Kit" according to manufacturer's instructions (BD Biosciences, Clontech). Hybridisation and chemiluminescent detection were carried out using the "SpotLight Chemiluminescent Hybridisation and Detection Kit" (BD Biosciences, Clontech). This method appeared to be less sensitive compared to radioactive detection.

### Real-time RT-PCR

Five different primer sets were designed within G72 exons that are common to most G72 splice variants (Exons 2, 4 and 7). Sequences are shown in table 1; G72 #2 corresponds to the primer pair used by Korostishevsky *et al*. [6]. All primer sets were tested by SYBR Green PCR (and TaqMan PCR, if applicable) on human genomic DNA (Clontech; 0.1 ng – 0.1 μg per reaction), human adult total brain, amygdala, testis and fetal brain cDNA (Marathon Ready cDNA, Clontech; 0.1 – 0.5 ng cDNA per reaction) and cDNA from HEK-293 cells overexpressing recombinant His-G72 cDNA (see below). Total RNA was isolated from HEK-293 cells (mock-transfected or overexpressing His-G72) as described above. Samples were reverse transcribed (in triplicate) into cDNA using the Omniscript RT-PCR kit (Qiagen). Briefly, for each 20 μl reverse transcription reaction, 300 ng RNA in a total volume of 12 μl was added to 8 μl RT-PCR mastermix (final concentrations/amounts: 1 μM Oligo-dT primer (Invitrogen), 0.5 mM each dNTP, 1× RT Buffer, 10 units RNase inhibitor (Invitrogen), and 4 units Omniscript Reverse Transcriptase). Negative controls were prepared as above, but without Reverse Transcriptase. Reactions were incubated for 60 min at 37°C. The masterplate was aliquoted into 20 optical 96-well plates using the Hydra-96 robot (Robbins Scientific, GRI). Taqman PCR was performed using an ABI 7700 Sequence Detection system (PE Applied Biosystems) as previously described [7]. Briefly, the PCR reaction mastermix contained a final concentration of 200 nM of each of the forward and reverse primers (see table 1; Invitrogen), 200 nM FAM-TAMRA probe (Qiagen, Operon), and 1× Quantitect master mix (Qiagen). For SYBR Green chemistry, 1× SYBR Green mastermix (Perkin Elmer) was used, and no probe was added to the reaction. The PCR amplification was performed for 40 cycles, consisting of 50°C for 2 min, 95°C for 10 min, 40 cycles of 95°C for 15 s and 60°C for 1 min. Standards were analysed on each plate using dilutions of genomic DNA. All measurements were performed in triplicate. The housekeeping gene cyclophilin was run as a control to ensure comparable amounts of cDNA in all wells. Ct values were converted into arbitrary units according to manufacturer's instructions (Applied Biosystems).

**Table 1:** Primer and probe sequences for G72 and cyclophilin

Primer name & location	Primer and probe sequence (F: forward, R: reverse, P: probe)
G72 #1 (Exon 4)	F: 5'-GGATGGAAGAGAAGGCATGAGG-3'
	R: 5'-TCTGCATAGGGCTGAGGAAGG-3'
	P: 5'-ATTTGGAAATGGCACAGAGG-3'

G72 #2 (Exon 7)	F: 5'-GCTGAATGAGTTTGGAAGCA-3'
	R: 5'-TGGGTCCCAGACACAGAGT-3'

G72 #3 (Exon 2)	F: 5'-CATTGGGTAAAATCTACTTCATAGG-3'
	R: 5'-GAGTTTAGAGAGTTTTCAGATTTGC-3'

G72 #4 (Exon 4)	F: 5'-GCATGAGGACGGCTATTTGG-3'
	R: 5'-TCTGCATAGGGCTGAGGAAGG-3'

G72 #5 (Exon 7)	F: 5'-CCGCAGGCAGCCTCTAGAAC-3'
	R: 5'-TCAGAAGGATTGGCTGGGAAG-3'
	P: 5'-CCAGCAAAAAGACCAGTCATGCAACCA-3'

Cyclophilin	F: 5'-TGAGACAGCAGATAGAGCCAAGC-3'
	R: 5'-TCCCTGCCAATTTGACATCTTC-3'
	P: 5'-AGCACCAATATTCAGTACACAGCTTAAAGCTATAGGTT-3'

Primers and probes for G72 Real-Time RT-PCR (SYBR^® ^Green and TaqMan^®^) and for the housekeeper gene cyclophilin are shown above. For exon numbering, compare Fig. 2B.

### Generation of rabbit polyclonal antibodies against G72

Rabbit polyclonal antibodies were generated by Cambridge Research Biochemicals (Billingham, UK) against two peptides within the predicted G72 variant AY138546 protein sequence (antibodies #1410 and #1411: antigenic peptide VTRKEGWKRRHEDGY-acid, with an N-terminal cysteine added for coupling; antibodies #1412 and #1413: antigenic peptide SKDRRQPLERMWTC-amide). Crude antisera were purified by affinity chromatography on Thiopropyl Sepharose 6B derivatised with the antigen. The concentration of purified antibodies in glycine eluates was as follows: #1410: 1.69 mg/ml, #1411: 0.96 mg/ml, #1412: 0.64 mg/ml and #1413: 0.33 mg/ml.

### Preparation of protein lysates

Transfected HEK-293 cells were washed twice with cold PBS and lysed in 500 μl – 1 ml ice-cold Triton-X-100 lysis buffer (50 mM Tris-HCl pH 7.5, 150 mM NaCl, 5% Glycerol, 1% Triton-X-100, supplemented with "Complete" Protease Inhibitor Cocktail (Roche) and phosphatase inhibitor cocktail (Sigma)) per 10 cm-plate. Samples were rotated for 15 min at 4°C and centrifuged 20 min at 14 000 g, 4°C. Supernatants were stored in aliquots at -80°C.

Rat tissue was dissected from embryonic, young or adult rat (CD, Charles River), ground to a powder in liquid nitrogen and stored at -80°C. Total protein lysates were prepared from tissue powder using RIPA lysis buffer (50 mM Tris-HCl pH 7.5, 150 mM NaCl, 1% NP40, 0.5% Na-Deoxycholate, 0.1% SDS, supplemented with "Complete" Protease Inhibitor Cocktail (Roche) and phosphatase inhibitor cocktail (Sigma)). Samples were tip sonicated briefly, rotated at 4°C for 15 minutes and centrifuged for 20 min at 14 000 g, 4°C. Supernatants were collected and protein concentration was determined using the BCA Protein Assay Reagent Kit (Pierce Biotechnology).

### Immunoprecipitation

Cell lysates from transiently transfected HEK-293 cells were prepared as described above, using 750 μl to 1 ml Triton-X-100 lysis buffer per 10 cm plate. Supernatants were incubated with 3 μg of anti-G72 antibody (#1410 or #1411) for 2 h at 4°C, followed by incubation with protein-G-sepharose (Sigma) for another 2 h at 4°C. Immunocomplexes were washed twice with lysis buffer and once with 1× TBS before elution with 40 μl of 2× Laemmli loading buffer containing β-Mercaptoethanol. Eluates were analysed by SDS-PAGE and Western blotting as described below.

### SDS-PAGE and Western blotting

Total lysates from transfected HEK-293 cells (lysate preparation see above; 10 – 20 μg total protein per well), rat tissue lysates (40 – 50 μg per well) or human protein medleys (50 μg per well; Clontech/BD Biosciences: SDS-solubilised proteins prepared from whole human tissues, prepared under conditions designed to ensure maximal representation of tissue-specific proteins) were separated on 4–20% Novex Tris-Glycine gels (Invitrogen). Proteins were transferred to nitrocellulose (Amersham) or PVDF (Millipore) membrane by wet-blotting. For conventional Western analysis, membranes were blocked with 5% non-fat milk powder in TBST (25 mM Tris pH 7.5, 150 mM NaCl, 0.1% Tween-20) for 1 h at room temperature, incubated overnight at 4°C with primary antibody (anti-G72 #1410, #1411, #1412 or #1413 at 1–5 μg/ml; anti-His H-15, goat polyclonal antibody, Santa Cruz, at 0.5 μg/ml; anti-actin, mouse monoclonal, ascites fluid, Chemicon, 1:20 000 dilution) in blocking buffer, washed five times in TBST, incubated with goat-anti-rabbit-IgG-POD (Santa Cruz, 1:10 000 in blocking buffer) for 1 h at room temperature, washed again and developed using the ECL Plus (Amersham), ECL Advance (Amersham) or Visualizer (Upstate) detection system. Alternatively, Western analysis was performed using the LI-COR Odyssey^® ^Infrared Imaging System. Experiments were performed according to manufacturer's instructions. Briefly, proteins were separated as described above alongside Odyssey pre-stained molecular weight markers (Licor, fluorescence in 700 nm channel) and transferred onto PVDF membrane (Millipore) by wet blotting. Membranes were blocked with Odyssey blocking buffer (LI-COR), diluted 1:1 with 1 × PBS. For primary antibody incubations, the blocking buffer was supplemented with 0.1% Tween-20, and for secondary antibody incubations, 0.1% Tween-20 and 0.01% SDS were added. Primary antibody concentrations and incubation times were as described above. Blots were washed five times with PBST (1 × PBS, 0.1% Tween-20) prior to a 1 h incubation (room temperature, protected from light) with secondary antibodies (IRDye800CW goat-anti-rabbit, LI-COR, 1:10 000; Alexa Fluor^® ^680 donkey-anti-goat, Molecular Probes 1:15 000). Membranes were washed again and visualised with LI-COR Odyssey^® ^Infrared Imaging System (700 nm and 800 nm channels).

### Immunocytochemistry

HEK-293 cells were seeded on 13 mm glass coverslips (coated with poly-D-lysine, Sigma), transiently transfected with FLAG-G72 or His-G72 cDNA and fixed with 4% paraformaldehyde in PBS (pH 7.2) for 15 min at room temperature 36 h post-transfection. Cells were blocked for 1 h at room temperature using PBS with 0.1% Triton X-100, 10% normal goat serum (NGS) and 1% bovine serum albumin (BSA). Following one wash with 1× PBS, cells were incubated overnight at 4°C with primary antibodies in PBS with 5% NGS and 0.5% BSA. Rabbit polyclonal anti-G72 #1410 was used at a concentration of 3 μg/ml, #1411 at 5 μg/ml and anti-FLAG M2 mouse monoclonal antibody (Sigma) at 0.3 μg/ml. After four 10 min washes with 1× PBS, cells were incubated for 1 h at room temperature with Alexa Fluor^®^488 goat anti-rabbit IgG and Alexa Fluor^®^594 goat anti-mouse IgG at 5 μg/ml (Molecular Probes). Cells were washed four times with 1× PBS, mounted with ProLong^® ^Gold antifade reagent (Molecular Probes) and analysed using an Olympus microscope and Image-Pro Plus imaging software (MediaCybernetics).

### Bioinformatics methods

#### Sequence information

Gene sequence information was obtained from the National Center for Biotechnology Information (NCBI) Genbank database. Variants AY138546 and AY138548 were used to represent G72 and G30 respectively. In early 2007, NCBI replaced AY138456 with a longer sequence, NM_172370.3, which is extended at the 5'UTR by 228 bp. This study will refer to AY138546 as the G72 variant; however, analyses have been repeated on NM_172370.3 for completeness.

#### G72 gene environment

The region around G72 was assessed using the UCSC human genome browser (March 2006; NCBI build 36.1). All known genes (with a Genbank RefSeq transcript) in a specified region can be displayed using this viewer.

#### Identification of Promoter regions

Promoters were predicted for G72 and G30 by collating and analysing results from several promoter prediction programs. Each of the features predicted by the programs adds a layer of confidence to the promoter prediction. The programs report CpG rich regions using EMBOSS newcpgseek (threshold CpG score of 17) & cpgplot, and CpGProD [1,8,9], nucleosomal binding sites using recon [10], transcription start sites using eponine [11], human transfac transcription factor site clusters using Cister [12], and EMBOSS matrix/scaffold attachment regions using marscan [13].

#### Identification of Functional motifs

Secondary structure elements and previously identified motifs present in a number of motif databases were scanned for using the protein sequences of G72 and G30. Twelve structure predictions were performed including transmembrane helix prediction TMHMM [14], and prediction/location of signal sequences SignalP [15]. Six motif databases were searched including a database of protein motif regular expressions and profiles PROSITE [16] and a database of protein fingerprints, each fingerprint usually consisting of multiple motifs PRINTS [17].

#### EST searches

An extensive EST search was carried out using BlastN run through the following subsets of Genbank: Genbank EST, Genbank cDNA Genbank Human EST and Genbank Rat EST against more than fifteen species. The default BLAST Expectation thresholds used were lowered to 100 to show weaker matches. In addition, using the NCBI blast tool, G72 (both AY138546 and NM_172370.3) and G30 transcript sequences were run through BLASTN against all of the available EST and cDNA databases.

#### Ortholog searches

To search for evidence of G72 or G30 orthologues, several Genbank databases were searched. The nucleotide transcripts sequences were run through BLASTN against all the (more than fifteen) species specific EST and cDNA GenBank subdivisions. The protein sequences were run through BLASTP against fourteen species specific GenBank protein databases. The sequences were also run through BLASTN against over fifteen general primate and rodent genomic sequence databases.

#### Identification of RNA instability motifs

The G72 UTRs were searched for predicted AU-rich elements (AREs) using the motif ATTTA [18].

#### Identification of miRNAs

Two miRNA prediction methods, miRANDA [19] and RNA hybrid [20] were run on the G72 transcripts to search for evidence that the gene is regulated by miRNAs. The thresholds for free energy of binding for miRANDA was -15, and RNA hybrid is -25. P values for both methods were < = 0.05.

#### Gene prediction

All of the gene prediction algorithms available as tracks in the UCSC (May 2004 version, NCBI build 35) were run on G72 and G30 sequences. The majority of the methods used to predict genes are based on gene models reconstructed solely from mRNA and EST evidence. Therefore in this analysis we have focused on prediction methods that also use evidence from other sources.

N-SCAN [21] combines biological-signal modelling in the target genome sequence along with information from a multiple-genome alignment to generate de novo gene predictions. Gene predictions were taken from the SGP program, which predicts genes using mouse/human homology [22]. The exoniphy program identifies evolutionarily conserved protein-coding exons in a multiple alignment using a phylogenetic hidden Markov model phylo-HMM [23], a statistical model that simultaneously describes exon structure and exon evolution. Retrogenes are predicted by showing processed mRNAs that have been inserted back into the genome since the mouse/human split [24]. RetroGenes can be either functional genes that have acquired a promoter from a neighboring gene, non-functional pseudogenes, or transcribed pseudogenes. The ExonWalk program merges cDNA evidence together to predict full length isoforms, including alternative transcripts. The Vertebrate Genome Annotation (VEGA) database build 30 is designed to be a central repository for manual annotation of different vertebrate finished genome sequence [25]. Finished genomic sequence is analysed on a clone by clone basis using a combination of similarity searches against DNA and protein databases as well as a series of *ab initio *gene predictions using GENSCAN [26]. In addition, comparative analysis using vertebrate datasets such as the Riken mouse cDNAs and Genoscope *Tetraodon nigroviridis *Ecores (Evolutionary Conserved Regions) are used for novel gene discovery.

The coding region of the chimpanzee G72 was predicted from chimpanzee genomic sequence, using human G72 protein sequence (NP_758958) as a template and the GENEWISE gene prediction tool [27].

## Results

### Detection of G72 by northern analysis

**Figure 1:**
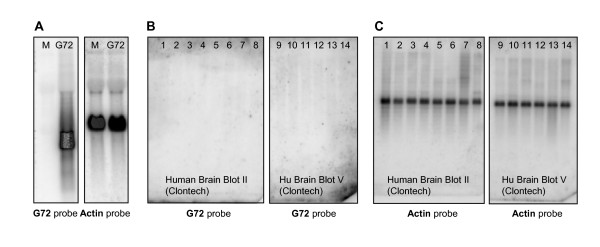
**Investigation of G72 expression by northern analysis**. (A) G72 and β-actin probes were validated using total RNA isolated from mock-transfected HEK-293 cells (M) or cells expressing His-G72 (G72). A strong signal for G72 was only observed in cells expressing His-G72. (B) Using a G72-specific, radioactive probe, no G72 signal was detected on Clontech poly A+ RNA Northern blots. The following brain regions were represented: Human Brain Blot II: 1, putamen. 2, temporal lobe. 3, frontal lobe. 4, occipital pole. 5, spinal cord. 6, medulla. 7, cerebral cortex. 8, cerebellum. Human Brain blot V: 9, thalamus. 10, whole brain. 11, hippocampus. 12, corpus callosum. 13, caudate nucleus. 14, amygdala. (C) Following G72-probing, human brain blots II and V were stripped and reprobed with a β-actin-specific probe in order to ensure RNA integrity and equal loading. Signal strength was similar in all lanes.

A G72-specific 390 bp probe was isolated by restriction digest from G72 cDNA clone AY138546 (plasmid His-G72, see methods), radioactively labelled and validated using northern blot membranes containing total RNA from HEK-293 cells overexpressing His-G72 (10 μg total RNA per lane). As shown in Figure 1A, a strong and specific signal was seen in His-G72 overexpressing cells, but not in mock-transfected cells. RNA from both His-G72 and mock-transfected cells showed a strong beta-actin signal (Fig. 1A, right). We next sought to investigate expression of G72 mRNA across a variety of human CNS regions, using Clontech MTN (Multiple Tissue Northern) human brain blots. The northern membranes contained 2 μg of poly A+ mRNA per lane, a quantity that should allow detection of rare messages. Probes were radioactively labelled, since initial experiments had shown a lower sensitivity of non-radioactively labelled probes (data not shown). In order to increase the probability of detecting potentially low levels of G72, washing conditions were kept at a low stringency (see Methods), and phosphorimager exposure times were increased to up to three days. Whereas a strong G72 signal was visible after 2 – 12 hours on the validation blots (Fig. 1A), no G72 mRNA was detected in any of the CNS regions tested, even after very long exposure times (Fig. 1B). These results were unexpected, since CNS regions represented on Clontech human brain blots II and V included amygdala (lane 14), caudate nucleus (lane 13) and spinal cord (lane 5), i.e. regions from which G72 has previously been cloned [1]. Lane 8 contained mRNA from cerebellum, a region that might be expected to express G72 if its role was indeed regulation of D-amino acid oxidase which is expressed at highest levels in this brain region [28,29]. However, this brain region was also devoid of detectable G72 mRNA. In order to control for RNA integrity and similar RNA loading in all lanes, G72-probed northern membranes were stripped and reprobed with a beta-actin probe. As shown in Fig. 1C, a strong actin signal was observed after short exposure times (2 – 6 hours). Thus, G72 levels in all brain regions investigated are below the detection limit of northern analysis.

### Analysis of G72 mRNA expression in brain using SYBR-Green and Taqman RT-PCR

**Figure 2:**
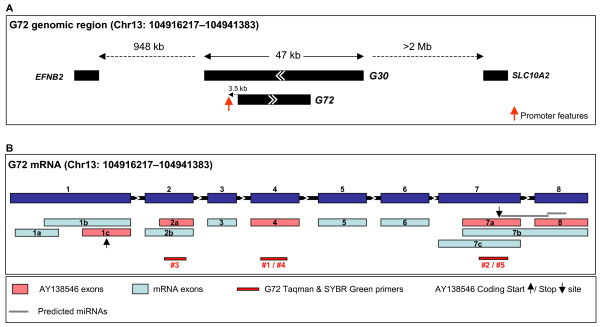
**Genomic context and gene structure of the G72 gene on Chr. 13**. (A) Representation of the genomic context around the G72 gene on chromosome 13, showing overlapping and neighbouring genes. The red arrow indicates the region where the in silico promoter tools predict features (see main text). (B) Gene structure of G72 showing exons of splice variant AY138546 in pink and exons of other variants in blue. G72 Taqman and SYBR Green primers are indicated in red, the AY138546 coding start and stop sites are shown as black arrows and predicted miRNA sequences in the AY138546 UTR are depicted in grey.

**Figure 3:**
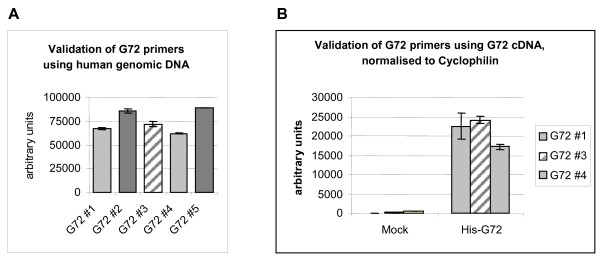
**Validation of G72 primer sets using real-time RT-PCR**. (A) Primer sets G72 #1, #2, #3, #4 and #5, which were designed within the most common G72 exons (see table 1 and Fig. 2B) were validated using human genomic DNA. Light grey: primer sequence within exon 4. Dark grey: primer sequence within exon 7. Hatched: primer sequence within exon 2. All primer sets gave a strong signal using human genomic DNA (A) and no signal using rat genomic DNA (not shown). (B) Primers sets within exon 2 (hatched) or exon 4 (light grey) readily detected a signal in cDNA that was isolated from HEK-293 cells expressing a His-tagged G72 cDNA, but not in mock-transfected cells (values within range of empty controls). The exon 7 reverse primer annealing sequence was not contained in our G72 cDNA construct and did thus not detect His-G72 (not shown).

In an attempt to employ a more sensitive detection method, we used real-time semiquantitative RT-PCR to analyse expression levels of G72 in human tissues and cell lines. Five primer sets (see table 1 in Methods) were designed within G72 exons that are common to most G72 splice variants (exons 2, 4 and 7; see Fig. 2B), including one primer pair (#2) previously used by Korostishevsky *et al*. [6]. Primer design within exons, rather than across exon-intron boundaries, allowed validation of primer sets using human genomic DNA (see Fig. 3). However, as the small size of all exons limited the possibilities for primer and probe design, SYBR Green chemistry (primer sets #2, #3 and #4) was used as an alternative to TaqMan RT-PCR (primer sets #1 and #5). The G72 genomic context, gene structure and positions of PCR primers and probes are shown in Fig. 2.

As shown in Fig. 3A, all five primer sets gave a robust signal on human genomic DNA. No signal was obtained using rat genomic DNA (data not shown), proving specificity of the primers for human G72, as the rat genome does not contain a G72 gene (see below, and [1]). However, none of the primer pairs detected significant levels of G72 mRNA in human cDNA from brain, testis, spinal cord or amygdala, i.e. regions from which G72 has reportedly been cloned. In addition, no G72 amplification could be achieved in cDNA from human fetal brain (Clontech) or in more than ten different human cell lines. Since it has previously been suggested that G72 might be upregulated in schizophrenic brain [6], we also investigated G72 expression in a carefully selected set of human post-mortem brain BA10 samples from more than 25 schizophrenic patients and matched controls (Charing Cross Hospital Prospective Collection). No expression of G72 mRNA could be detected in either control or schizophrenic brain (random hexamer primed cDNA: data not shown). This result is in contradiction to a previously published report [6], but is consistent with other unreported results (e.g. Dr. Phil Burnet, University of Oxford: personal communication using the Stanley Foundation Samples).

In order to verify that our primer sets were able to amplify G72 from cDNA, and not only from genomic DNA, we prepared cDNA from HEK-293 cells expressing a His-tagged version of the longest G72 ORF, the coding region of variant AY138546 (His-G72). Only primer sets within exon 2 and 4 (#1, #3, #4) were expected to give a signal using His-G72 cDNA. The reverse primers of primer sets in exon 7 (#2 and #5) were designed to anneal to the untranslated 3'-UTR region which was not contained within our expression plasmid. However, the 3'-UTR is reportedly present within the splice variants described in GenBank and thus primer sets in exon 7 were expected to work on human cDNA samples. As shown in Fig. 3B, primer sets within exons 2 and 4 readily amplified G72 cDNA from G72-overexpressing cells, thus confirming that our primers were able to detect G72 cDNA when mRNA is expressed at appreciable levels. Real-time semi-quantitative PCR is a highly sensitive technique that we and others have previously used to amplify rare messages. Our results therefore suggest that endogenous expression levels in adult human total brain, testis, spinal cord, amygdala, BA10, or human fetal brain are below the detection limit for TaqMan/SYBR Green PCR and must thus be extremely low, if present.

### Generation and validation of G72 antibodies

**Figure 4:**
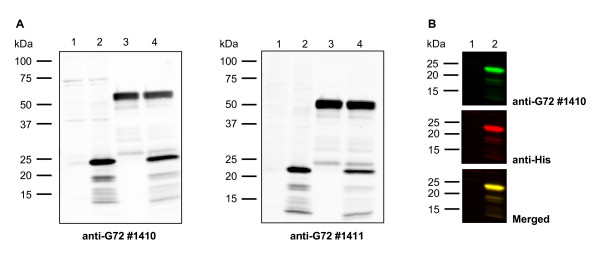
**Validation of anti-G72 antibodies using western blotting and LI-COR Odyssey^® ^Infrared Imaging System**. (A) Western blotting was performed using rabbit polyclonal antibodies anti-G72 #1410 (left) or anti-G72 #1411 (right). Lane 1: total protein lysate from mock-transfected HEK-293 cells. Lane 2: total protein lysate from His-G72-transfected HEK-293 cells. Lane 3: Eluate from immunoprecipitation (IP) of mock lysate with anti-G72 #1411. Lane 4: Eluate from IP of His-G72 lysate with anti-G72 #1411. A strong signal for His-G72 was detected at ~22 kDa (lanes 2 and 4), with weaker bands around 18 kDa and below. Additional bands in lanes 3 and 4 (weak signal at 25 kDa and strong signal at 50 kDa) represent the light and heavy chain of the antibody used for IP. (B) G72-specific antibody #1410 (upper panel, green) and a commercial anti-His antibody (middle panel, red) detect the same protein bands on Western blots (merged image, lower panel, yellow) in total protein lysates from HEK-293 cells overexpressing His-G72. Lane 1 & lane 2: as described in A.

Four antibodies against two G72 peptides were raised in rabbits. Anti-G72 #1410 and #1411 were directed against amino acids 55–69, #1412 and #1413 against amino acids 119–132 of the predicted G72 variant AY138546 protein, pLG72. Antibodies were validated using western blotting and immunoprecipitation. N-terminally His-tagged G72 (His-G72) was overexpressed in HEK-293 cells and total protein lysates or eluates from immunoprecipitation reactions were analysed by western blotting, using mock-transfected cells as a control. As shown in Fig. 4A, both #1410 and #1411 readily detected His-G72 in cell lysates (lane 2), but showed no specific signal in mock-transfected cells (lane 1). The apparent molecular weight (Mw) of His-G72 was ~22 kDa, which is similar to the observed Mw of *in vitro *translated G72 (~24 kDa, [1]) and slightly heavier than the predicted Mw of ~18 kDa. The observed size difference is likely to be due to the epitope tag. Several lower molecular weight bands were observed and are likely to be degradation products of His-G72, since they were detected by anti-G72 antibodies but not seen in mock lysates. Both antibodies also immunoprecipitated His-G72 from cell lysates, as shown for antibody #1411 (Fig. 4, lanes 4; analogous data for immunoprecipitation with #1410 not shown). In order to demonstrate that the observed protein at ~22 kDa was indeed His-tagged G72, membranes were probed with anti-G72 and anti-His antibodies and visualised using LI-COR Odyssey^® ^Infrared Imaging System, which allows simultaneous detection of two antibodies. As shown in Fig. 4B (lane 2), the anti-G72 antibody #1410 (green) and a commercial anti-His antibody (red) detected the same bands in total protein lysates from HEK-293 cells overexpressing His-G72 (merged image in yellow). Analogous results were obtained using antibody #1411 (data not shown).

Antibodies #1410 and #1411 were also validated for immunocytochemistry. Both polyclonal antibodies specifically stained G72-transfected, but not mock-transfected HEK-293 cells. G72 showed a mainly punctate expression pattern (Additional File 1), which is typical for mitochondrial proteins and which paralleled the subcellular G72 localisation recently described by Kvajo *et al*. [3]. Antibodies #1412 and #1413 did not yield a specific signal in western blotting or immunocytochemistry experiments and did not bind G72 in IP reactions. Therefore, only antibodies #1410 and #1411 were used in future experiments.

### Attempted detection of G72 protein in human brain lysates

**Figure 5:**
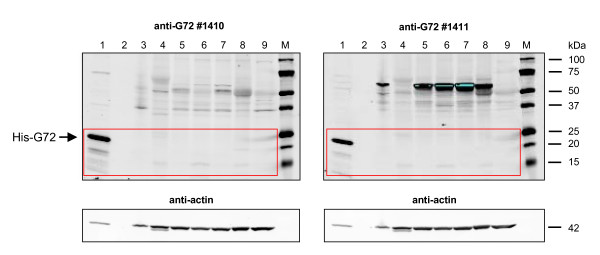
**Attempted detection of G72 protein in human samples by western blotting**. Western blot membranes were probed with anti-G72 #1410 and #1411 as indicated above and visualised using the LI-COR Odyssey^® ^Infrared Imaging System. Lane 1: total protein lysate from His-G72-transfected HEK-293 cells (positive control). 2: no sample. 3–8: Clontech/BD Biosciences human "protein medleys"; 3: spinal cord. 4: testis. 5: amygdala. 6: cerebellum. 7: frontal lobe. 8: fetal brain. 9: total protein lysate from rat cortex, postnatal day 14 (negative control – no G72 gene in rat). M: molecular weight standards. No specific signal at the expected size of ~18 kDa was detected in any of the human samples. A very weak signal at ~15 kDa was seen in testis, cerebellum and fetal brain (lanes 4, 6, 8). Anti-G72 #1411 detected a strong unspecific signal of unknown identity in all human CNS samples (lanes 3, 5, 6, 7, 8). Blots were reprobed with anti-actin as a loading control (lower panel).

Detection of endogenous G72 protein in human tissues reportedly expressing G72 [1,5] was attempted using human protein lysates ("protein medleys", BD Biosciences, Clontech) from seven different CNS regions (spinal cord, amygdala, cerebellum, frontal lobe, fetal brain, hippocampus, temporal lobe) and testis. His-G72-containing lysates were used as a positive control in all experiments. In order to detect even weak signals, a variety of very sensitive detection reagents as well as LI-COR Odyssey^® ^Infrared Imaging was used. Representative results are shown in Fig. 5. No specific signal for endogenous G72 was observed at the expected molecular weight of ~18 kDa with either of the previously validated antibodies, anti-G72 #1410 and #1411. An unspecific band just above 50 kDa was observed with #1411 in all human CNS samples (lanes 3, 5, 6, 7, 8), but not in testis (lane 4) or rat brain (lane 9). A very weak band, the visibility of which could be increased through scanning at higher intensity (analogous to longer exposure times), was observed at around 15 kDa (i.e. below the expected size for human G72) in testis (lane 4), cerebellum (lane 6) and fetal brain (lane 8), but not in spinal cord, amygdala, frontal lobe or rat cortex.

When western blots were developed using the very sensitive detection reagent "ECL Advance" in combination with long exposure time (> 10 min), anti-G72 antibody #1410 detected a signal at around 20 kDa, i.e. slightly above the expected size for human G72, in the human fetal brain protein medley (Additional File 2). This band was not detected using antibody #1411. Furthermore, a signal at the same size was also observed in rat cortex, although rodent species do not possess a G72 orthologue, thus making it unlikely that the observed signal in fetal brain represented endogenous G72.

Taken together, our western blotting experiments with a range of sensitive detection methods did not detect native G72 protein in a variety of human brain regions (including cerebellum and amygdala), spinal cord or testis.

### *In silico *analysis of G72 transcription

We performed a detailed *in silico *analysis of the G72/G30 locus to try and identify known motifs and regulatory elements that may give some insight into our inability to detect any G72 expression. As reported previously, the genomic region around G30 and G72 is devoid of genes with the nearest neighbouring known gene located 948 kb upstream (EFNB2) and over 2 Mb downstream (SLC10A2) of AY138546 (Fig. 2A). The G72 gene contains eight exons (Fig. 2B); the G72 splice variant AY138546 spans 24.8 kb and NM_172370.3 spans 25.0 kb. G72 is antisense to G30 (putative protein 10.4 kD) and AY138548, which represents the longest G30 variant, spans 47 kb.

*In silico *promoter prediction was performed on the genomic region spanning 5 kb upstream and 0.5 kb downstream of the gene transcriptional start site (TSS) of the G72 variant AY138546. This revealed a weak Cister peak (see Methods) indicating human transcription factor site clusters about 0.7 kb upstream of the TSS but no other prominent promoter features, such as CpG islands or cognate transcription factor binding sites which are often seen when a canonical promoter is present. However, there were some features consistent with a functional promoter (based on the prediction of CpG rich regions by the EMBOSS newcpgseek program, transcription factor site clusters by Cister and matrix/scaffold attachment regions by marscan) in the region 3.5 kb upstream of the TSS, although an EST database search revealed no ESTs (see below) or mRNAs that extended exon 1 of the G72 transcript 3.5 kb upstream. Although it is still possible that the regulatory region is located 3.5 kb upstream of G72 or that the G72 promoter is atypical, our analysis does not support the presence of a robust promoter.

In order to analyse G72 expression, a comprehensive EST database search was performed using all G72 variants. This failed to detect any significant G72 ESTs in any of the species (more than fifteen) searched providing no supporting evidence of expression. Furthermore, using human mRNA and protein G72 sequences along with predicted primate G72 protein sequences, searches of *Pan troglodytes *(chimpanzee), *Macaca mulatta *(rhesus monkey), *Macaca fascicularis *(cynomolgus monkey) and several other primate databases did not detect any ESTs or non human mRNAs or proteins. This strongly suggests no expression of G72 in these species in the tissues represented in the EST databases (or that it is only expressed in very specific libraries). Difficulties in detecting substantial G72 expression could potentially be accounted for by unstable mRNA species. The RNA instability motif search run on the G72 UTRs predicts one ATTTA motif in the 5'UTR of G72. These motifs are generally found in unstable mammalian mRNA UTRs and represent the most widespread and efficient determinant of RNA instability among those characterized in mammalian cells, marking RNAs for rapid destruction unless protected [30].

The G72 transcript sequence was also analysed by two miRNA prediction programs in order to investigate if it could be regulated by miRNAs. Using both methods, two (hsa-mir-600 and hsa-mir-196a-2) miRNAs predicted to bind to the 3'UTR (exon 7a and 8 of G72, see Figure 5B, corresponding to the 4^th ^and 5^th ^exon of the AY138546 variant) were identified. Although these programs predict miRNA target sites, the binding of the miRNAs to G72 would depend on other factors. For example, both the miRNA and transcript must be expressed in the same tissue at the same time and the folding of the mRNA UTR could affect accessibility of the miRNA to the binding site. For this reason it would be important to experimentally confirm these *in silico *predictions.

## Discussion

The G72/30 locus has received considerable attention due to replicated disease association data for schizophrenia and bipolar disorder. Furthermore, it has been proposed that the G72 peptide is a direct modulator of DAO activity and as such has mechanistic relevance to schizophrenia.

In order to better understand the function of G72, we investigated expression of both the G72 mRNA and putative protein in a series of human CNS and peripheral tissue samples. We were unable to detect the G72 message using either Taqman or northern analysis (13 brain regions) in any of our human tissue samples including cerebral cortex, amygdala, caudate nucleus, spinal cord and testis, i.e. tissues from which G72 had previously been cloned [1,5]. We also analysed over 25 control and 25 schizophrenic patient BA10 samples and could not detect the G72 transcript in the control or disease samples. Furthermore, we generated two specific and sensitive antibodies to a G72 peptide (amino acids 55–69 of LG72). Although the recombinant protein was readily detected by western blotting and immunocytochemistry, native protein was not observed in different tissues reportedly expressing G72.

Several gene detection methods that combine DNA/protein database searches and comparative analysis of conserved vertebrate DNA sequences predict the G72 gene at the locus on human chromosome 13q.33. Our in silico analysis, however, indicates that the G72 gene lacks features of a robustly transcribed gene and moreover contains an instability sequence that is generally found in unstable mammalian mRNAs [31]. There is also evidence for the presence of miRNA cognate sites, which regulate the expression of target genes by binding to their mRNAs [20]. These findings suggest that the G72 transcript may be rapidly removed if or when it is expressed. Furthermore, endogenous RNA interference between G72 and G30 (which is transcribed in the opposite direction) may also account for very low or no detectable expression, as suggested previously [2].

There is some evidence that G72 is only present in higher primates and not in rodent species [1]. Extensive genomic sequence analysis identifies some regions of homology to human G72 in *Pan troglodytes *(chimpanzee), *Gorilla gorilla *(gorilla), *Pongo pygmaeus *(orangutan), *Hylobates sp*. (gibbon), *Macaca mulatta *(rhesus monkey) and *Callithrix jacchus *(marmoset) genomic sequences. There are, however, no full-length primate orthologues and for example, there are two stop codons in the predicted chimpanzee G72 transcript sequence, suggesting this could in fact be a pseudogene and furthermore that AY138546 (and NM_172370.3) is unique to humans.

Although we (and others: Dr. Phil Burnet, University of Oxford, personal communication) have been unable to detect G72 mRNA, it has been possible to clone G72 from several libraries [1,3,5]. Indeed, we have cloned G72 cDNA from a testis cDNA library, but nevertheless failed to detect appreciable levels of G72 from the same testis cDNA sample using TaqMan or SYBR Green RT-PCR. Since these methods can detect tens of copies of a gene, expression levels below this detection limit are indicative of either no expression within any of the investigated tissues, or of an extremely localised or tightly regulated expression.

A significant percentage of human mRNA transcripts are either not polyadenylated or are bimorphic [32]. Although we failed to detect G72 transcripts in polyadenylated mRNA samples (northern analysis; real-time semiquantitative RT PCR analysis using cDNA from cell lines or Clontech "Marathon Ready cDNA") we also did not detect G72 transcripts in the human post mortem samples that were prepared with random hexamers, which excludes the possibility that our analysis may have failed to detect G72 transcripts based on the method of cDNA synthesis from isolated RNA. A possible reason why e.g. Korostishevsky et al. [6] observed a G72 PCR product may be explained by the apparent lack of sample treatment to eliminate contaminating genomic DNA and the absence of RT minus PCR controls for signal detection. We used the same primer pair as described by that group (primer set #2) and furthermore confirmed activity on genomic DNA (Fig. 3). It would seem likely therefore that the discrepancy between that study and our study (and others) might be explained by amplification from contaminating genomic DNA.

It should be noted that a recent study also reports difficulty in identifying native G72 protein and failed to replicate the proposed modulation of DAO activity [3]. The authors claim, however, to detect the native protein in a small (0.01%) sub-population of HeLa cells and in the membrane fraction of human amygdala. We have also analysed G72 expression (mRNA and protein) in the human amygdala and found no detectable G72 present. The antibody generated in the present study is very similar to that described by Kvajo *et al*. [3] in that we used the same epitope to generate the antibody and the cellular localisation of recombinant G72 is also punctuate/tubular rather that Golgi-restricted as suggested by Chumakov *et al*. [1] (Additional File 1). We also detected recombinant G72 by western analysis but not the native protein in tissue or cell extracts. When combining a very sensitive western blot detection method and long exposure times, a signal at around 20 kDa, i.e. just above the expected size of human G72 (18 kDa) was detected in human fetal brain lysate (Additional File 2). However, this signal was only observed with one of our two validated anti-G72 antibodies and a signal at the same apparent molecular weight was also detected in brain lysates from rat, which does not have a G72 gene, thus making it highly unlikely that this band represents G72. These results highlight the importance of suitable negative controls, as well as the danger of potentially misinterpreting bands if technical limits of the method are pushed (e.g. through loading of large protein quantities, long exposure times and high antibody concentration).

While further studies are required to understand the significance of the G72/30 locus to schizophrenia, we propose that if native G72 protein exists at all, it is expressed at such low levels that any physiological role is called in to question. We also conclude that the lack of demonstrable G72 expression in relevant brain regions does not support a role for G72 protein in modulation of DAO activity and the pathology of schizophrenia via a DAO-mediated mechanism. Following the original proposal for a direct G72 and DAO interaction [1], the interaction has not been confirmed using an endogenous tissue source. More importantly, two recent reports have failed to confirm the previous findings [3,33], which is consistent with our own inability to reproduce the protein-protein interaction of G72 and DAO using recombinantly expressed proteins (data not shown).

Whilst we were unable to detect G72 expression in human fetal brain, it remains a possibility that G72 is developmentally regulated and plays a unique spatio-temporal role in human brain development, independent of an interaction with DAO. We have not had the opportunity to investigate this further.

## Conclusion

Robust genetic findings from several independent studies have suggested the G72/G30 locus as a common susceptibility region for schizophrenia and bipolar disorder. This study investigated the mRNA and protein expression of the G72 gene product in human tissues and aimed to shed light on the physiological role of the predicted G72 protein, which has thus far not been resolved. Our experimental results, supported by an *in silico *analysis, do not provide any evidence of appreciable G72 expression in human brain or other human tissues at either the mRNA or the protein level. Thus, native G72 protein, if expressed at all, appears to only be present at extremely low levels that may not be physiologically relevant. This casts doubt on the proposed G72 protein modulation of DAO activity, which has been suggested to contribute to the pathology of schizophrenia. Further studies are required therefore to understand the significance of the G72/30 locus to schizophrenia.

## Competing interests

The authors declare that they have no competing interests.

## Authors' contributions

IB: Experimental design, conducted all experiments that contributed to Figures 1, 3, 4, 5 and Additional files 1 &2, analysis of results, writing of manuscript, discussion of experimental results. JNCK: concept and interpretation of study, writing of "background" paragraph, manuscript revision. RV: *in silico *analysis of G72 expression and writing of the respective manuscript sections (Bioinformatics methods and results). THC: Cloning of epitope-tagged G72 eukaryotic expression vectors. FK: QPCR analysis of CCHPC samples. JB and SH: Collection of CCHPC samples and discussion of experimental results. PRM: concept and interpretation of study, discussion of experimental results, writing of manuscript, manuscript revision.

All authors read and approved the final manuscript.

## Pre-publication history

The pre-publication history for this paper can be accessed here:



## Supplementary Material

Additional file 1 **Subcellular localisation of G72 protein in HEK-293 cells.** This figure shows the mainly punctate expression pattern of recombinant G72 in transfected HEK-293 cells, which is typical for mitochondrial proteins. (A) Transfection with FLAG-G72, staining with anti-G72 #1410 (upper panel) or anti-G72 #1411 (lower panel) and anti-FLAG M2. (B) Transfection with His-G72, staining anti-G72 #1410 (upper panel) or anti-G72 #1411 (lower panel). Click here for file

Additional file 2 **Attempted detection of G72 protein in human fetal brain and testis.** Western blots with Clontech "human protein medleys" (lane 1: fetal brain, lane 2: testis) and total protein lysate from rat cortex, postnatal day 14 (negative control; lane 3). Probed with anti-G72 #1410; developed using "ECL Advance" and long exposure time (> 10 min). A signal at around 20 kDa, i.e. only slightly above the expected size for human G72, was detected in human fetal brain and rat brain. However, a signal at the same size was also detected in rat brain (lane 3). Since rodents to not possess a G72 orthologue (see main text), the observed signal at 20 kDa appears to be unspecific, rather than representing endogenous G72 expression. Click here for file
