# Supplementary figures and images for: Investigation of G72 (DAOA) expression in the human brain
Source: BMC Psychiatry. 2008 Dec 11;8:94. doi: 10.1186/1471-244X-8-94 (PMC2630984; doi:10.1186/1471-244X-8-94)

## Slide 1
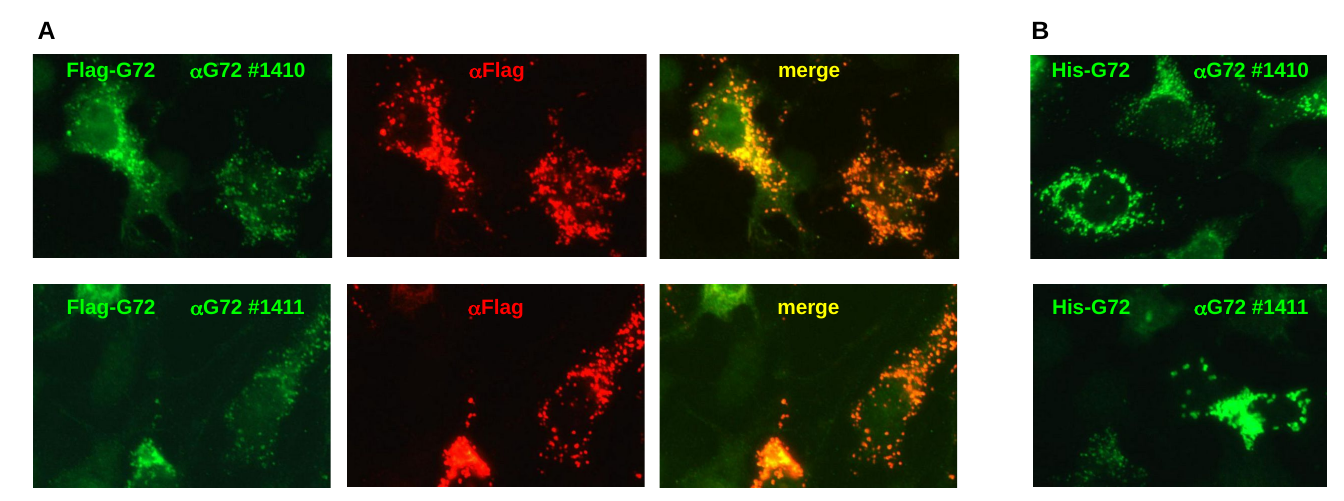

A
B
Flag-G72 αG72 #1410
αFlag
merge
His-G72 αG72 #1410
αFlag
Flag-G72 αG72 #1411
merge
His-G72 αG72 #1411

Supplement: Additional file 1 — Subcellular localisation of G72 protein in HEK-293 cells. This figure shows the mainly punctate expression pattern of recombinant G72 in transfected HEK-293 cells, which is typical for mitochondrial proteins. (A) Transfection with FLAG-G72, staining with anti-G72 #1410 (upper panel) or anti-G72 #1411 (lower panel) and anti-FLAG M2. (B) Transfection with His-G72, staining anti-G72 #1410 (upper panel) or anti-G72 #1411 (lower panel). [file 1471-244X-8-94-S1.ppt]

## Slide 1
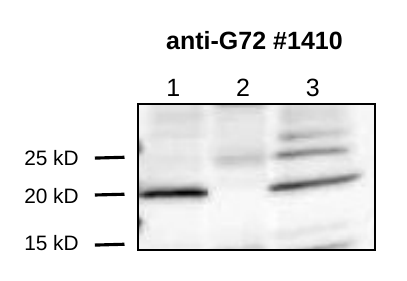

anti-G72 #1410
 1 2 3
25 kD
20 kD
15 kD

Supplement: Additional file 2 — Attempted detection of G72 protein in human fetal brain and testis. Western blots with Clontech "human protein medleys" (lane 1: fetal brain, lane 2: testis) and total protein lysate from rat cortex, postnatal day 14 (negative control; lane 3). Probed with anti-G72 #1410; developed using "ECL Advance" and long exposure time (> 10 min). A signal at around 20 kDa, i.e. only slightly above the expected size for human G72, was detected in human fetal brain and rat brain. However, a signal at the same size was also detected in rat brain (lane 3). Since rodents to not possess a G72 orthologue (see main text), the observed signal at 20 kDa appears to be unspecific, rather than representing endogenous G72 expression. [file 1471-244X-8-94-S2.ppt]
